# The suppression of scale-free fMRI brain dynamics across three different sources of effort: aging, task novelty and task difficulty

**DOI:** 10.1038/srep30895

**Published:** 2016-08-08

**Authors:** Nathan W. Churchill, Robyn Spring, Cheryl Grady, Bernadine Cimprich, Mary K. Askren, Patricia A. Reuter-Lorenz, Mi Sook Jung, Scott Peltier, Stephen C. Strother, Marc G. Berman

**Affiliations:** 1 Keenan Research Centre of the Li Ka Shing Knowledge Institute, St. Michael’s Hospital, 30 Bond St, Toronto, ON, M5B 1W8, Canada; 2 Rotman Research Institute, Baycrest Hospital, 3560 Bathurst St, Toronto, ON, M6A 2E1, Canada; 3 Department of Medical Biophysics, University of Toronto, 101 College St, Suite 15-701, Toronto, ON, M5G 1L7, Canada; 4 School of Nursing, University of Michigan, 426 N Ingalls St, Ann Arbor, MI, 48104, USA; 5 Department of Psychology, University of Michigan, 400 N Ingalls St, Ann Arbor, MI, 48109, USA; 6 Department of Radiology, University of Washington, 4245 Roosevelt Way NE, Seattle, WA, 98105, USA; 7 College of Nursing, Chungnam National University, Jung-gu, Munhwa-ro 266, Daejeon, 301-747, South Korea; 8 Biomedical Engineering, University of Michigan, 2200 Bonisteel Blvd, Ann Arbor, MI, 48109, USA; 9 Institute of Medical Science, University of Toronto, 1 King’s College Cir, Toronto, ON, M5S 1A8, Canada; 10 Department of Psychology, University of Chicago, 5848 S University Ave, Chicago, IL, 60637, USA; 11 Grossman Institute for Neuroscience, Quantitative Biology and Human Behavior, 5812 S Ellis Ave, Chicago, IL, 60637, USA

## Abstract

There is growing evidence that fluctuations in brain activity may exhibit scale-free (“fractal”) dynamics. Scale-free signals follow a spectral-power curve of the form *P*(*f* ) ∝ *f*^*−β*^, where spectral power decreases in a power-law fashion with increasing frequency. In this study, we demonstrated that fractal scaling of BOLD fMRI signal is consistently suppressed for different sources of cognitive effort. Decreases in the Hurst exponent (*H*), which quantifies scale-free signal, was related to three different sources of cognitive effort/task engagement: 1) task difficulty, 2) task novelty, and 3) aging effects. These results were consistently observed across multiple datasets and task paradigms. We also demonstrated that estimates of *H* are robust across a range of time-window sizes. *H* was also compared to alternative metrics of BOLD variability (SD_BOLD_) and global connectivity (Gconn), with effort-related decreases in *H* producing similar decreases in SD_BOLD_ and Gconn. These results indicate a potential global brain phenomenon that unites research from different fields and indicates that fractal scaling may be a highly sensitive metric for indexing cognitive effort/task engagement.

Fractalness is a ubiquitous property of nature. This scale invariant, self-similar property is used to describe the growth of trees, the formation of mountains, the branching of blood vessels and the crashing of ocean waves. Fractals, as noted by Benoit Mandelbrot (a pioneer in the field of fractal geometry), “are present everywhere.” Fractals are created by repeating a simple process in a recursive way that produces the same pattern over different scales. For example, consider a snowflake’s spatial pattern. As you zoom in on the snowflake with a microscope, the same pattern will be seen as you increase the magnification. Fractalness occurs not only in the spatial domain, but also in the temporal domain, and has been used extensively in the study of brain function^1^,^2^,^3^,^4^,^5^,^6^,^7^,^8^,^9^,^10^,^11^. Here, we build on this seminal work, and in particular the work of Barnes, Bullmore & Suckling^5^ and He and colleagues^6^,^7^, and hypothesize that brain signals exhibit more “fractal” properties in the time domain when the brain is in a more rested state, and therefore exerting less effort.

For temporal signals, measures of fractalness (or scale invariance) determine whether a specific time-scale or frequency plays a predominant role in cognitive processes; for scale-free signals, all examined time-scales/frequencies contain significant information. Scale-free signals exhibit a power-law relationship, where the spectral power at frequency *f* takes the form *P*(*f* ) ∝ *f*^*−β*^ for scalar coefficient β > 0. This coefficient is related to the Hurst exponent^12^ (H) via the equation β = 2H-1, with a higher value of *H* indicating more scale-free signal. Fractal processes exhibit long-range dependence in time, meaning that the signal at a given time-point is likely to be similar to preceding time-points, leading to more “smooth-looking” time-series fluctuations.

Many properties of brain function are associated with scale-free signals. Neuronal spike trains have a 1/*f* distribution^13^, and electroencephalography (EEG) has a scale-free, broadband signal^1^ that is correlated with behavioural measures^2^ and frequency-band analyses^3^. These properties extend to blood oxygen level dependent functional magnetic resonance imaging (BOLD fMRI), as Van De Ville and colleagues^4^ have established that BOLD signal can be modeled as a sum of scale-free EEG “microstates”. Importantly, and most relevant to our hypothesis, is the work of Barnes and colleagues^5^ and He and colleagues^6^,^7^ who demonstrated that the Hurst exponent is highest during rest, and decreases during tasks. Additionally^14^, and^15^ demonstrated that *H* is inversely correlated with reaction time (RT). There has also been significant research extending beyond standard fractal analysis of brain signals, including models of scale-free inter-regional connectivity^9^ and multifractal analysis, in which brain signals are associated with a spectrum of scaling exponents^8^,^16^,^17^. Inspired by prior work, which demonstrated that fractal scaling of BOLD signal is increased at rest and modulated by task performance^5^,^6^,^7^, we hypothesize that fractal scaling decreases globally as a function of cognitive effort.

Effort is not a trivial concept to quantify, but behavioural metrics are often used in an attempt to measure effort, including speed (i.e., reaction time) and accuracy^18^,^19^,^20^,^21^. Nonetheless, behavioural performance and effort are not always equivalent. For example, “compensatory effort” involves maintaining behavioural performance under fatigue and distraction^18^. Physiological measures are potentially more sensitive, including blood glucose and cardiac function, pupil dilation, muscle tension, and adrenaline levels^18^,^22^,^23^, although they are only indirect markers of the underlying neuronal processes implicated in effort. To address this gap, BOLD fMRI may provide a more direct measure of the relationship between effort and brain function. Standard fMRI analyses during task engagement show increased activation in task-specific brain regions with greater effort^23^,^24^,^25^. Conversely, effort may reduce activity in task-specific regions and the default-mode network^23^,^25^,^26^. However, standard fMRI analysis methods only quantify mean BOLD activity, and treat fluctuations about the mean as noise, thus neglecting potential effort-related changes in BOLD dynamics.

Task difficulty, task novelty and aging involve common and unique demands, which can be construed as increasing cognitive effort. For example, task difficulty, task novelty and aging all typically involve increased reaction time and decreased accuracy^27^,^28^,^29^,^30^, which are often construed as behavioural metrics of cognitive effort. This is particularly true of reaction time, as trial-by-trial differences in reaction time have been associated with fluctuations in cognitive effort and level of preparation^30^. Reaction time may be an even stronger correlate of cognitive effort when accuracy is equalized across task difficulty, novelty and aging, as this would reduce any strategic changes, which may include speed-accuracy tradeoffs.

This conceptualization of cognitive effort is related to seminal work by Shiffrin and Schneider, who distinguished between controlled vs. automatic processing^31^,^32^. Controlled processing is highly demanding of attentional resources, is thought to reflect serial processing, and is strongly dependent on cognitive load^32^. This mode of processing is different from automatic processing, which is relatively well learned in long-term memory, is potentially more parallel in nature and is much less affected by cognitive load^32^. Tasks that are not well-practiced, and that vary on load/difficulty, are associated with more effortful controlled processing and subsequently have increased reaction time and decreased accuracy. Similarly, we expect that older adults require more controlled processing compared to younger adults when performing tasks where accuracy levels are high, as in our data. While numerous definitions of “cognitive effort” exist, we believe it to be a useful characterization as a psychological factor that is increased across these experimental manipulations.

In this study, we used fractal-scaling measures to quantify changes in brain dynamics and their relation to effort. We focused on three sources of cognitive effort that have wide relevance in fMRI studies: task difficulty, task novelty, and aging (older- vs. younger-adults). We hypothesized that each source of effort would push the brain into a less fractal state (Fig. 1). We also examined the link between fractal scaling and two other commonly used measures of BOLD dynamics, the standard deviation of BOLD signal (SD_BOLD_) and global functional connectivity (*Gconn*). BOLD variability has been previously shown to correlate with *H* despite being statistically unrelated^6^, and similarly, BOLD variability and global functional connectivity are significantly related^33^. Moreover, both metrics have been linked to cognitive functioning and aging^34^,^35^,^36^. It is therefore critical to understand the link between these metrics and scale-free BOLD dynamics. In summary, we hypothesize that BOLD fractal scaling provides a consistent, robust correlate of cognitive effort across a range of experimental manipulations.

## Results

Scale-free or “fractal” scaling requires signals to be statistically invariant over a range of timescales. This requires that signal fluctuations do not predominate at any specific time-scale (or frequency). For this paper, the Hurst exponent (*H*) was calculated as an index of fractal scaling at each brain voxel. In principle, power spectral density (PSD) may be used to calculate *H*, by measuring the slope β of a linear fit between the log-transformed values of spectral power *P*(*f* ) and frequency *f*, and computing *H* = (*β* + 1)/2. However, the PSD approach is highly sensitive to low frequency trends, which may be a significant confound in BOLD fMRI. As an alternative, we used Detrended Fluctuations Analysis (DFA). This technique measures the power in BOLD fluctuations *F*(*n*) as a function of time windows of length *n*. The slope α of a linear fit between the log-transformed *F*(*n*) and *n* is equal to *H*, with α = 1 indicating a perfectly fractal signal. This approach is more robust to trends in the data, which may otherwise confound fractal-scaling estimates.

**Figure 2:**
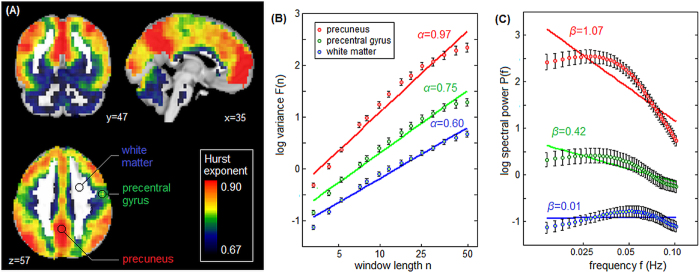
Representative Hurst exponent (*H*) scaling plots for selected brain regions. (**A**) average Hurst exponent map for Sustained Attention to Response Task (SART), averaged over all subjects, with selected regions of interest (ROIs) in the precuneus, precentral gyrus and white matter, as representative regions with high/moderate/low *H*. (**B**) average variance *F*(*n*) vs. time window size *n*, as estimated for Detrended Fluctuations Analysis (DFA), along with the slope of a linear fit α. (**C**) average spectral power *P*(*f* ) vs. frequency *f*, along with the slope of a linear fit β. Results for (**B,C**) and linear fits are shown in log-log scale. Error bars represent Bootstrapped 95% confidence intervals (95%CIs).

The comparison of DFA and PSD is shown in Fig. 2, for a representative dataset. Figure 2A depicts a brain map of *H* values averaged across subjects, and Fig. 2B shows DFA analysis plots of log-transformed *F*(*n*) vs. *n*, for regions with high *H* (precuneus), moderate *H* (precentral gyrus) and low *H* (white matter). The fitted slope α is lower in the latter brain region, as expected. Similarly, Fig. 2C shows scaling curves obtained using PSD, which produces similar trends across the regions of interest. For both models, the data points show some curvature around the line of best fit, potentially indicating variability in *H* across time windows. However, the DFA values generally show a high degree of fit, with a coefficient of determination (*R*^2^) of 0.95 for the precuneus, 0.97 for the precentral gyrus and 0.97 for white matter. Conversely, PSD curves show lower fits, with *R*^2^ of 0.78 for the precuneus, 0.77 for the precentral gyrus and 0.67 for white matter, which is consistent with this method’s greater sensitivity to non-stationary signal. See *Methods: fractal scaling in fMRI* for further discussion of the relationship between PSD estimation and DFA.

To test the hypothesis that *H* is suppressed for different modulators of cognitive effort, we examined four different datasets: a block-design adaptation of the Trail-Making Test [TMT^37^], a fast event-related Sustained Attention to Response Task [SART^38^], an event-related verbal working memory task [VWMT^39^,^40^], and a block-design multi-task battery [MTAS^25^] consisting of different experimental condition blocks. These datasets were selected to cover a wide range of different cognitive tests and experimental designs. This allowed us to test the generalizability of the hypothesized relationship between BOLD dynamics and cognitive effort. All datasets showed a high degree of fit with the DFA model, with longer task runs producing better model fits. Across subjects, we measured mean whole-brain *R*^2^ of 0.91 ± 0.02 (MTAS), 0.95 ± 0.01 (TMT), 0.94 ± 0.01 (SART) and 0.98 ± 0.01 (VWMT). Supplementary Figure S1 plots the average *R*^2^ brain map of each task for both PSD and DFA Hurst estimators, demonstrating that DFA obtains better model fit for all datasets. Moreover, the *R*^2^ goodness of fit is highly consistent over all brain voxels for DFA, whereas PSD obtains higher fit in some regions (e.g., cingulate cortex, parietal lobes) and lower fit elsewhere (e.g., sensorimotor cortex).

The MTAS dataset was used to show that fractal scaling varies as a function of task complexity (*Results: Effects of Task Complexity*). Importantly, these data involve a set of tasks with consistent stimulus and response formats, but varying levels of complexity and difficulty. To evaluate the effects of task novelty (*Results: Effects of Task Novelty*), we measured the change in fractal scaling as a function of task exposure for TMT and SART, by comparing run-1 vs. run-2 for a single scanning session. We also used the multi-run VWMT data to show that fractal scaling increases continually as a function of task exposure. In other words, when the task is novel it is more effortful, and becomes more fluent with practice. We next examined the effects of aging on fractal scaling (*Results: Effects of Aging*), by comparing young and old adult groups for TMT and SART. We also used the VWMT data, which spans a continuum of ages, to show that fractal scaling decreases continually with age. All of the above analyses are performed using DFA to estimate *H*. In addition, the analyses are replicated using the PSD estimator (Supplementary Figures S2–S4), to determine whether the results depended upon the algorithm used to estimate *H*.

We also examined whether results are significantly impacted by the choice of the largest scaling window *n* for DFA (*Results: Impact of Scaling Window Size*). In the final results section, we compared *H* to two other widely used measures of BOLD dynamics, the standard deviation of BOLD signal (SD_BOLD_) and global functional connectivity (*Gconn*) (*Results: Relation to BOLD Variability and Functional Connectivity*). See *Discussion* for a comparison of fractal scaling results against standard contrast-based fMRI analyses.

### Effects of Task Complexity

We measured the effects of task complexity on fractal scaling using data from the MTAS battery. For each run of the battery, subjects performed 4 different tasks, separated by fixation blocks. All tasks required subjects to identify band-pass filtered white noise patches. We used 4 task conditions, which are ordered from most complex to most simple: a perceptual matching task (PMT); a delayed-match to sample recognition task (DMS); an attentional cueing task (ATT); and a reaction time task (RT). We observed increased accuracy and decreased response time for the simpler tasks (Table 1), which is consistent with an interpretation of increasing cognitive effort.

**Figure 3:**
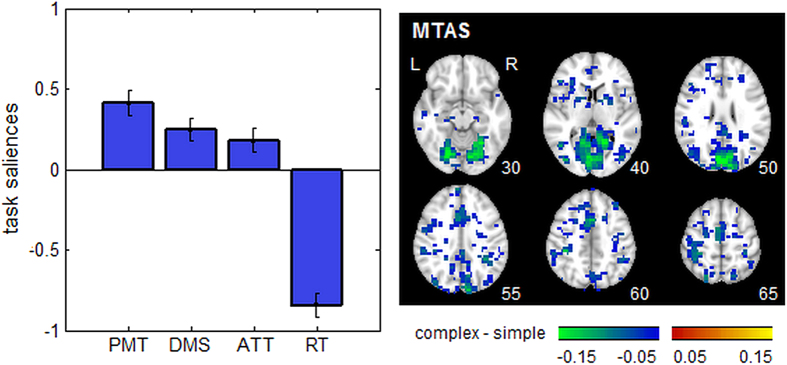
Brain regions showing decreased Hurst exponent (*H*) going from simple to complex tasks. Results of a Partial Least Squares analysis are shown for four different tasks obtained from a Multi-Task Assessment battery, reaction time (RT), attentional cueing (ATT), delayed match to sample (DMS) and perceptual matching (PMT). We display results of the first component, which accounts for 44.3% total covariance (significant at *p* < 0.001, permutation testing). (left) plots task saliences with Bootstrapped 95% CI errorbars, and (right) plots associated significant bootstrap ratio values in the brain at a False Discovery Rate threshold of 0.05, indicating widespread negative change in Hurst exponent, associated with the more complex DMS and PMT conditions. A liberal cluster-size threshold of >3 contiguous voxels was also applied to improve image interpretability.

Figure 3 shows a task Partial Least Squares (PLS) analysis, measuring how *H* varies parametrically across the 4 tasks, with bootstrap ratios indicating the reliability of implicated brain regions. For all analyses of cognitive effort, brain maps were thresholded at a conservative False-Discovery Rate (FDR) of 0.05 to control against spurious activations. Only one latent variable had significantly stable task saliences (*p* < 0.001 eigenvalue significance under permutation testing; accounting for 44.3% cross-block covariance). Task saliences show a graded effect of task complexity (left panel; PMT > DMS > ATT > RT) that is associated with greater reductions in Hurst exponent (right panel; negative Bootstrap values). This effect appears to be primarily localized to the regions implicated in visual-motor functioning, including the upper cerebellum, occipital lobes, motor cortex, inferior parietal lobes and supplementary motor area. This demonstrates that increasing differences in task difficulty lead to larger, more spatially extensive decreases in *H*. Supplementary Figure S2 shows the same analysis using a PSD estimator of *H*, where a similar graded effect of task complexity (PMT > DMS > ATT > RT) is obtained. However, the extent of significant brain voxels is greatly reduced compared to DFA.

### Effects of Task Novelty

**Figure 4:**
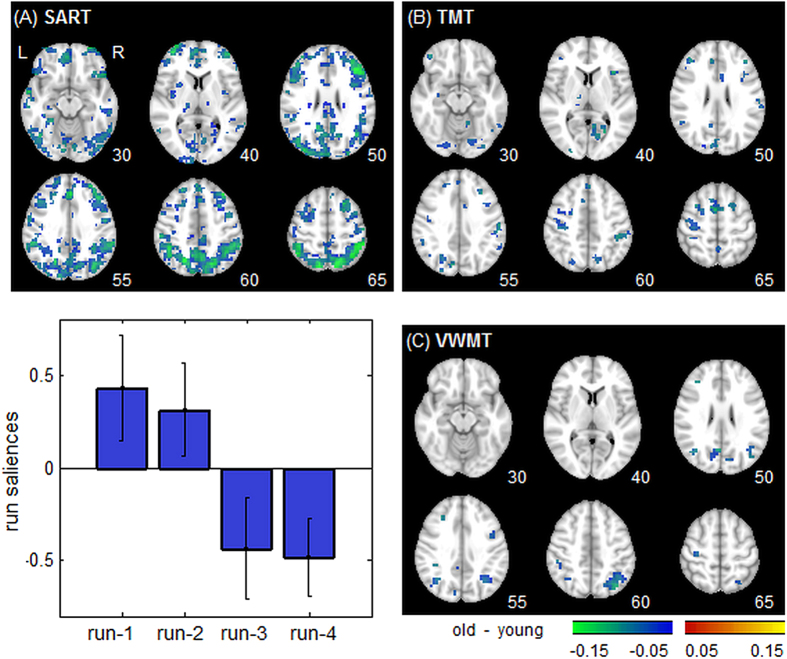
Brain regions showing decreased Hurst exponent (*H*) going from run-2 to run-1 of a task (increasing task novelty). Panels (**A,B**) show results of pairwise testing of Hurst exponent in run-1 vs. run-2, for TMT = Trail-Making Test (block design) and SART = Sustained Attention to Response Task (fast event-related). (**C**) Results of a Partial Least Squares analysis of 4 runs of a VWMT = Verbal Working Memory Task (slow event-related). We display results of the first component, which accounts for 54.0% total covariance (significant at *p* < 0.001, permutation testing), showing decreased Hurst exponent (negative Bootstrap ratios) is associated with later task runs. All Bootstrap ratio maps are corrected for multiple comparisons at FDR = 0.05 threshold; run saliences have 95% CI errorbars. A liberal cluster-size threshold of >3 contiguous voxels was also applied to improve image interpretability.

To test the effects of task novelty on fractal scaling, we compared run-1 (more novel) vs. run-2 (more familiar or practised) within a single scanning session of the SART, TMT tasks (Fig. 4A,B); we also evaluated fractal scaling across 4 consecutive scanning runs in VWMT (Fig. 4C). Here, task novelty is defined as inversely related to task exposure. Behavioural measures of Table 2 shows changes consistent with decreased cognitive effort in run-2; subjects performed significantly better during run-2 for TMT response time (*p* < 0.01; nonparametric paired Wilcoxon test), SART percent correct (*p* < 0.01) and VWMT response time (*p* = 0.04). The changes were non-significant for SART response time (*p* = 0.36) and VWMT percent correct (*p* = 0.19), but were in the predicted direction.

Figure 4A,B displays regions of significant change in *H* for SART and TMT, comparing task run-1 to run-2. Plots show widespread reduced fractal scaling in the more novel task run-1 relative to run-2 (i.e. negative Bootstrap ratios). Figure 4C shows a task Partial Least Squares (PLS) analysis, measuring how *H* varies across 4 consecutive runs of the VWMT task; as in the previous section, only one latent variable had significantly stable run saliences (*p* < 0.001 eigenvalue significance under permutation testing; accounting for 53.0% cross-block covariance). Run saliences show a continuous increase for earlier runs (left panel; run1 > run2 > run3 > run4), that is associated with decreased Hurst exponent (right panel; negative Bootstrap values). All three tasks show decreased *H* in the cuneus, precuneus, right angular gyrus, parietal lobe and supramarginal gyrus, along with dorsolateral prefrontal cortex. These results indicate that for a more novel, effortful presentation of the task, there is both decreased behavioural performance and reduced fractal scaling. Supplementary Figure S3 shows the above analysis for *H* estimated via PSD, where similar decreases in *H* are obtained for the more novel task.

### Effects of Aging

**Figure 5:**
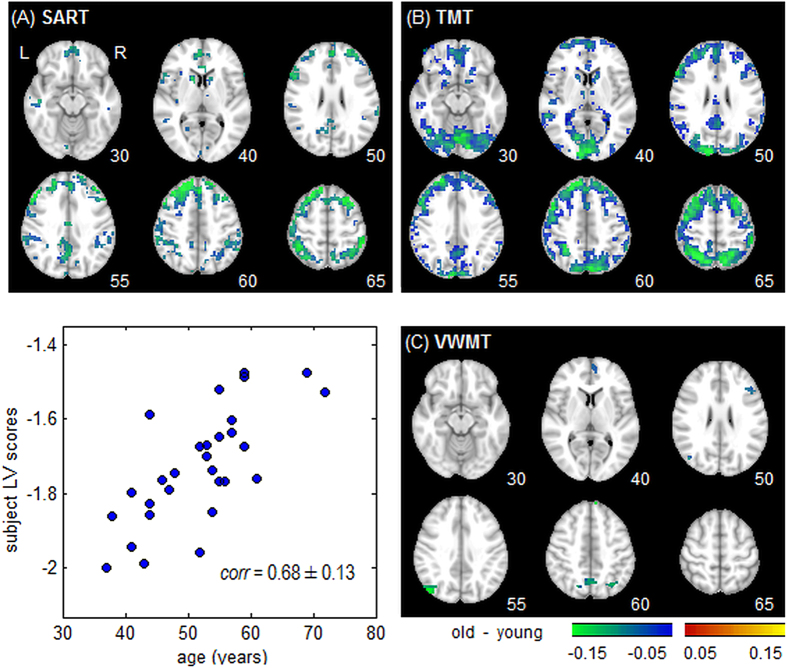
Brain regions showing decreased Hurst exponent (*H*) going from older to younger subjects (increasing age). Panels (**A,B**) show results of pairwise testing of Hurst exponent in young vs. old, for TMT = Trail-Making Test (block design) and SART = Sustained Attention to Response Task (fast event-related). (**C**) Results of a Partial Least Squares analysis of Hurst exponent against age for VWMT = Verbal Working Memory Task (slow event-related), showing decreased Hurst exponent (positive Bootstrap ratios) is associated with age. All Bootstrap ratio maps are corrected for multiple comparisons at FDR = 0.05 threshold. A liberal cluster-size threshold of >3 contiguous voxels was also applied to improve image interpretability.

To evaluate the effects of aging on fractal scaling, we compared younger vs. older groups for the SART and TMT tasks (Fig. 5A,B) where median age for the younger subjects was 24 (range 20–33) and for older subjects was 68 (range 61–82); we also looked at fractal scaling as a function of age in VWMT, which included a wide distribution of ages between 36 and 71 years old (Fig. 5C), as a more challenging analysis. Table 3 shows changes in behaviour consistent with increased cognitive effort for the older adults, comparing young vs. old groups in SART and TMT, and the 10 youngest vs. 10 oldest subjects of VWMT data; older adults had significantly reduced performance (p < 0.01 for all tasks, nonparametric Wilcoxon rank-sum test).

Figure 5A,B displays regions of significant change in H for SART and TMT, comparing task performance for older and younger participants. The plots show widespread reduced fractal scaling in the older subject group relative to the younger (i.e. negative Bootstrap ratios). Figure 5C shows a task Partial Least Squares (PLS) analysis, measuring how *H* covaries with age in the VWMT task, which only produces a single latent variable (i.e. age was the only behavioural regressor). Increased age is associated with significant decreases in Hurst exponent (left panel; negative Bootstrap values). All three tasks show decreased *H* in the posterior cingulate, cuneus, precuneus, and left middle frontal lobe. The larger young vs. old contrast of SART and TMT showed more extensive reductions in *H*, and more extensive changes in superior frontal, inferior frontal and ventromedial prefrontal regions. These results demonstrate that aging reliably produces decreased *H* across tasks, and for large differences in age, this occurs in a consistent set of brain regions. Supplementary Figure S4 shows the above analysis for *H* estimated via PSD, which also demonstrates decreasing *H* for the older aged cohorts.

### Impact of Scaling Window Size

**Figure 6:**
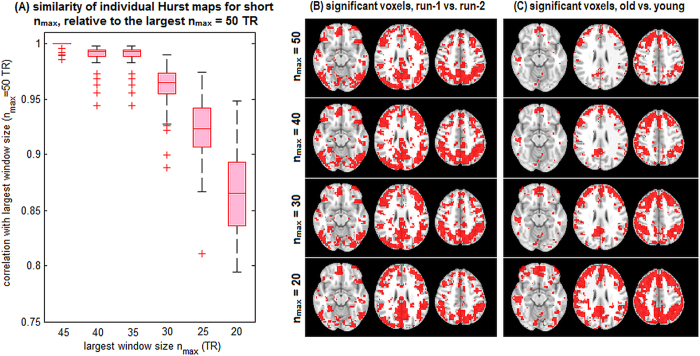
The impact of maximum scaling window size on DFA results. (**A**) boxplots plots showing correlations between individual subject *H* maps obtained for maximum window size *n*_max_ = 50 TR, compared to *H* maps obtained for shorter *n*_max_. (**B**) plots showing brain regions with significantly reduced *H* as a function of *n*_max_, for run-1 vs. run-2 (task novelty) and old vs. young (aging). Results are shown for the representative SART dataset.

For all analyses except task complexity, BOLD scaling phenomena were examined across a fixed range of 3 to 50 TRs. Due to the shorter block lengths of the MTAS data, *H* was computed for task complexity analyses from 3 to 20 TRs (6 to 40 sec). In this section, we examined whether *H* analyses of other datasets show consistent results at shorter *n*_max_, in order to validate its use in MTAS, and to determine if window size selection has a significant impact on the relationship between *H* and effort. Figure 6 depicts results for the representative SART dataset. Figure 6A plots the average correlation of each subject’s *H* map obtained from shorter *n*_max_, relative to the *H* map obtained for the largest window size *n*_max_ = 50. The patterns are nearly-identical down to *n*_max_ = 30 (median correlation > 0.95) and still show high consistency at *n*_max_ = 20. Figure 6B plots brain regions with significantly decreased *H* associated with task novelty, as a function of *n*_max_. The patterns are consistent, with *n*_max_ = 50 and *n*_max_ = 20 having a Jaccard overlap of 0.41. Figure 6C plots brain regions showing significantly decreased *H* associated with age, as a function of *n*_max_. Patterns are again relatively consistent, with *n*_max_ = 50 and *n*_max_ = 20 having a Jaccard overlap measure of 0.54. In summary, the maximum window size does not appear to significantly limit our ability to detect associations between *H* and cognitive effort.

### Relation to BOLD Variability and Functional Connectivity

**Figure 7:**
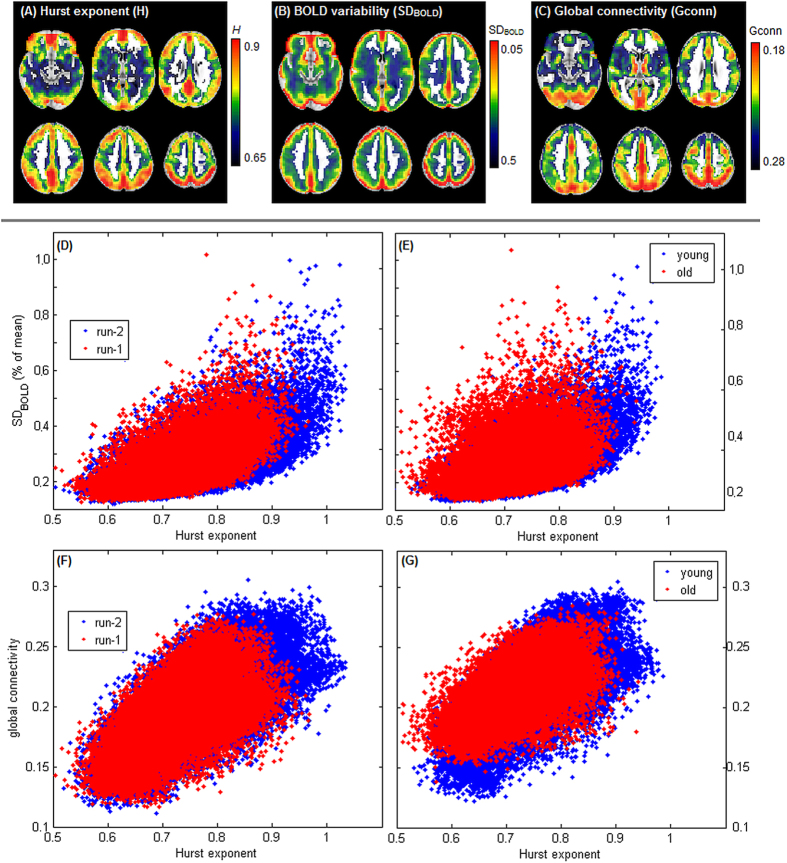
The relationship between Hurst exponent (*H*), BOLD variability (SD_BOLD_) and global connectivity (*Gconn*). (**A–C**) average brain maps computed across subjects, for *H*, SD_BOLD_ and *Gconn*. Scatterplots (**D,E**) show median *H* vs. SD_BOLD_ of brain voxels, for run-1 vs. run-2 (young subjects only) and old vs. young subject (averaged over runs). Scatterplots (**F,G**) show median *H* vs. *Gconn* of brain voxels, for run-1 vs. run-2 (young subjects only) and old vs. young subject (averaged over runs). Results are shown for the representative SART dataset.

For this section, the representative SART dataset was used to examine how *H* is related to two other widely used measures of BOLD dynamics, including BOLD variability (SD_BOLD_) and global connectivity (*Gconn*). Figure 7A–C shows the brain maps of these measures, averaged over all young subjects. The maps show some similarity, with high values mainly in posterior dorsal brain regions, although there are also specific differences. In particular, *H* tends to give higher weights near the orbitofrontal and anterior cingulate cortex, while SD_BOLD_ gives higher weights near the precuneus and brain edges, and *Gconn* gives relatively high weights in the occipital lobe and superior frontal brain regions.

Figure 7D–G displays scatterplots of median voxel values for *H* against SD_BOLD_ and *Gconn*, comparing run-1 vs. run-2 for young adults (Fig. 7D,F) and comparing old vs. young, averaged over both runs (Fig. 7E,G). As previously shown, voxels with highest *H* tend to be reduced in run-1 vs. run-2, and old vs. young, and a similar decrease is seen for SD_BOLD_ and *GConn*. Based on non-parametric comparison of distributions (Kolmogorov-Smirnov test), all metrics are associated with significant shift in values between run-1 and run-2, and between old and young cohorts (*p* < 0.001, for all tests). Comparing run-1 and run-2, *H* is associated with the largest shift in values, with a mean K-S statistic of 0.22 [95%CI: 0.11, 0.33], compared to SD_BOLD_ [K-S = 0.09; 95%CI: 0.03, 0.14] and GConn [K-S = 0.17; 95%CI: 0.04, 0.34]. Similarly, when comparing old and young cohorts, *H* is associated with the largest shift in values [K-S = 0.20 95%CI: 0.05, 0.36], compared to SD_BOLD_ [K-S = 0.08; 95%CI: 0.03, 0.17] and GConn [K-S = 0.17; 95%CI: 0.07, 0.36]. Nonetheless, the only significant difference was between *H* and SD_BOLD_ for run effects (run 1 vs. run 2; *p* = 0.001, Bootstrapped p-value), with all other comparisons non-significant at *p* > 0.13. Thus, *H* shows the greatest mean effect of effort, although SD_BOLD_ and *Gconn* also show significant effects, and no metric consistently shows significantly greater sensitivity to cognitive effort, though H is numerically larger.

We also examined the relative similarity of the brain maps estimated by these different metrics, summarized in Table 4. In general, *H* showed high correlations with bold SD_BOLD_ and *Gconn*, while *GConn* and SD_BOLD_ showed weaker correlations. In addition, Table 4 demonstrates that the strength of the association between these metrics is also decreased under higher cognitive effort. For all paired correlations, Run-1 shows lower correlations relative to Run-2, and older adults show lower correlations compared to younger adults. The most dramatic change is seen for old vs. young comparisons, with non-overlapping bootstrapped 95%CIs. Therefore, not only does cognitive effort suppress all three metrics, but it also reduces the strength of their association.

## Discussion

This paper tested the hypothesis that cognitive effort is reliably associated with decreased fractal scaling throughout the brain, by measuring changes in *H* for three different sources of cognitive effort: task complexity, task novelty, and aging. As hypothesized, increased cognitive effort was associated with a reliable decrease in the Hurt exponent of BOLD signal, with strong generalizability across multiple tasks and robust findings across a range of time-scales. This indicates that cognitive effort systematically alters BOLD dynamics throughout the brain, producing a scale-limited signal that is less “smooth” (i.e., more rapidly-varying fluctuations) and reduced long-range correlations.

Although this is the first study to examine changes in *H* across multiple tasks and sources of effort, our findings are consistent with previous studies of fractal scaling in fMRI. There have been multiple comparisons of *H* between resting and task-based fMRI, demonstrating that *H* tends to be higher at rest^5^,^6^,^7^, and that the lowest frequencies of BOLD signal show the greatest suppression during task engagement^41^, which is consistent with reduced *H*. In addition, *H* is was found to be inversely correlated with response time in the right inferior frontal cortex, for a face encoding task^17^. Fewer studies have examined other modulators of task difficulty, although^42^ established that low-frequency BOLD power increases over time during resting-state, which may lead to increased *H*, and is potentially due to increased subject drowsiness. In addition^15^, reported *increased H* with age during resting-states. While seemingly contradictory with our current findings, the results from^15^ may actually support our hypothesis that decreases in *H* with age are driven by effort (not age alone), because *H* does not increase with age during non-effortful resting states.

For task difficulty (Fig. 3), the change in *H* is primarily localized to the cuneus and visual cortex, indicating that it is primarily the dynamics of visual processing that are altered. These regions only partially overlap with active areas from standard fMRI task analyses. For example, strong task contrasts in MTAS (PM vs. RT and AT vs. RT) show decreased activation in the cuneus and superior occipital lobe, but the predominant signal changes included decreased activation in the posterior cingulate, and increased activation in the inferior parietal lobe^43^. Moreover, these changes in task activation are bi-directional (i.e. with regions of positive and negative change), thus the direction of activation change is not a reliable indicator of task-related effort. In contrast, the change in *H* is primarily uni-directional, which has the advantage of not requiring *a priori* knowledge of implicated brain regions when using *H* to index cognitive effort.

For task novelty (Fig. 4), we used an inverse measure of task exposure; as participants gain experience with the task, we observed both improved behavioural performance and increased fractal-scaling *H*. A set of brain regions is reliably implicated in task exposure, including the posterior cingulate, the inferior frontal lobe and the middle frontal lobe. As with *task difficulty*, task activation studies of early motor adaptation (i.e. adapting to task novelty) report changes in brain regions that partially overlap with our findings, but with bi-directional changes in activity: decreased parietal and striatal activation, and increased motor, premotor and cerebellar activation^44^. This again demonstrates that uni-directional change in *H* can reliably index task habituation.

For aging effects (Fig. 5), the decrease in *H* is most extensive in dorsal regions associated with attention, but significant changes are also observed in sub-regions of the default mode network including the posterior cingulate cortex and the ventral anterior cingulate cortex. We can compare these results with prior task activation studies^25^, where greater activity was reported in the task-positive network (including inferior parietal and dorsolateral prefrontal cortices) and reduced activity in the default-mode network (including posterior cingulate, ventral anterior cingulate and middle temporal lobes) with age. Thus, there is considerable overlap in brain regions, but as with *task difficulty* and *task novelty*, the changes in *H* are broadly uni-directional, whereas for standard fMRI activation analyses, the directionality depends on differing brain regions.

We also found that the DFA algorithm produces values of *H* that are associated with effort for time-series as brief as 20 TRs. This is demonstrated in the analysis of task difficulty, which was conducted on relatively short blocks of 20TRs, and a subsequent analysis of SART data (Fig. 6), which showed consistent results for a maximum window size *n*_max_ ranging from 50 to 20 TRs. This agrees with fractal scaling theory, as scale-invariance implies consistent scaling properties regardless of the analyzed range. Nonetheless, fMRI data are often limited by finite sample sizes and significant moment-to-moment changes in BOLD dynamics, requiring validation for this approach. Our results support the analysis of *H* over brief time periods, and indicate that findings are robust to analyzed window size.

In a series of supplementary analyses, we also replicated our analyses of task-related changes in *H* with a PSD-based estimator of fractal scaling. For both DFA and PSD estimators, we report decreased *H* during conditions of greater cognitive effort, which further supports the generalizability of our findings. This is also aligned with He *et al.*^6^, who showed consistent findings for both estimators. However, the set of significant brain regions differs, with the greatest difference in brief task blocks of the MTAS data, where DFA detects much more extensive brain regions than PSD. While a previous study found the PSD approach to be more robust than DFA for fMRI data^45^, our findings suggest that this advantage may depend on the cognitive task and number of time-points being analyzed. In addition, the PSD estimator consistently shows lower mean *R*^2^ model fit, and more spatial heterogeneity of model fit. Taken together, these findings suggest that the effects of cognitive effort on *H* are reliable, but the DFA model provides a more robust fit for shorter time windows of fMRI data.

We also examined the relationship between *H* and other widely used measures of resting-state brain dynamics (Fig. 7), including BOLD variability (SD_BOLD_) and global functional connectivity (*Gconn*), showing that *H* is spatially correlated with SD_BOLD_ and *Gconn*, and these metrics are also reduced during cognitive effort. SD_BOLD_ quantifies the effective “dynamic range” of neurovascular functioning at each voxel, and correlations with *H* have been previously observed^6^. The present findings provide further evidence that higher BOLD dynamic range may be an important feature allowing brain regions to express scale-free dynamics. *GConn* quantifies total integration at each voxel, and its correlations with fractal scaling may indicate that *H* is a marker of highly functionally connected brain regions. The present findings are consistent with literature showing reduced functional connectivity and *H* during task performance, relative to resting-state^9^. Interestingly, the strength of the associations between *H*, SD_BOLD_ and *Gconn* was also shown to be affected by cognitive effort, with more effortful conditions showing lower correlation between metrics. This further underscores the complexity of changes in brain dynamics during cognitive effort, which may not be fully described by a single metric.

There are a number of potential limitations to the current study, as well as considerations for future work. Due to the focus on cognitive effort, the current study is necessarily based on task data. One important question is how this relates to resting-state; for example, are older adults consistently less scale free, or only under overt task performance? Prior research has shown that scale-free dynamics are significantly correlated with subjective states of self-consciousness while at rest^46^, indicating that robust relationships between brain dynamics and cognition can be extracted during this state. A second issue is that it may be important to understand how these findings relate to other cognitive factors, such as psychological and physical distress. We have recently shown that fractal scaling is suppressed under high anxiety and low fatigue in breast cancer patients^40^. In the current framework, this suggests that anxiety may invoke similar brain dynamics as cognitive effort, provided one has sufficient energy to engage in worrying, which may further extend our conception of brain dynamics and cognitive effort.

Another important area of future investigation is whether the current findings generalize to other functional neuroimaging modalities. As the current findings related to scale-invariance and generalize across a range of window sizes, we might expect them to also be manifested in more rapid electrophysiological processes in the brain. This is supported by the work of Van De Ville and colleagues^4^, who found that fMRI signal can be well-described as a function of scale-free EEG microstates. Given this promising link, future research should examine the effects of cognitive effort on EEG signal, with an emphasis on microstate dynamics.

Finally, the current analyses assumed that the data exhibit a single scaling exponent over all time scales. This has the advantage of computational efficiency and leads to simpler, more interpretable results. While the linear slope fitting of DFA produced a high degree of fit across all measured time-scales (with average *R*^2^ > 0.90 for all datasets), when examining Fig. 2 there appears to be some curvature about the fitted line, potentially indicating variability in *H* across time-scales. Termed “cross-over” phenomena^47^, this has been previously observed in biological data, and may indicate the presence of multiple scaling regimes in fMRI data. Similarly, DFA is a “mono-fractal” technique, which assumes that data are Gaussian distributed, and scaling is fully defined by variance (the second-order moment). This may be an over-simplified representation of a richer fractal scaling structure, defined across multiple statistical orders, which can be assessed using “multi-fractal” analysis^8^,^15^. Further studies will be required to determine if cognitive effort is related to different mono-fractal scaling regimes, and higher-order multi-fractal scaling exponents.

Our results show that effort, from a number of different sources, systematically decreases fractal scaling throughout the brain, pushing the brain into a more limited dynamic range state. Conversely, when effort is not exerted, the brain stays in a highly fractal state, with greater dynamic range. In the future, this metric may also be extended to characterize the relationship of BOLD signal with other sources of effort, including cognitive decline associated with disease and brain injury, and provide a useful metric in the clinical domain as well. We have begun to examine this already in breast cancer patients^40^. In particular, fractal scaling measures may be used to assess cognitive effort for complex, ecologically-valid tasks where it is often difficult or impossible to obtain simple summary measures of behaviour, including passive attending, visualization tasks, creative tasks and mental arithmetic. In summary, we find that fractal scaling may be a unifying metric for quantifying cognitive effort across many and varied sources.

## Materials and Methods

### Experimental fMRI Data and Preprocessing

We examined 4 datasets, obtained from 3 different fMRI studies, in order to demonstrate the generalizability of scale-free phenomena. This included a Multi-Task Assessment battery (MTAS), the Trail-Making Test (TMT), a Sustained Attention to Response Task (SART), and a Verbal Working Memory Test (VWMT). All datasets exhibited generally low motion ranges, estimated based on the median absolute displacement relative to the mean, and thus no subjects were excluded from analysis. Examining inferior-superior motion, which was the axis of greatest displacement for all subjects, we observed a mean of 0.07 ± 0.07 for MTAS (max. 0.28), 0.14 ± 0.10 for TMT (max. 0.42 mm), 0.08 ± 0.07 mm for SART (max. 0.48  mm) and 0.05 ± 0.03 mm for VWMT (max. 0.09 mm). The details of each dataset and associated pre-processing are described below. The preprocessing pipelines were chosen to be consistent with prior task-based analyses on each respective fMRI dataset, in order that current results could be directly compared with these findings in the Discussion.

#### MTAS Dataset

This dataset included 19 young, healthy adults (median age 25 years, range 20–30, 8 women), screened using a health questionnaire to exclude health issues. Images were acquired with a Siemens Trio 3-T magnet. T2 functional images (TE/TR = 30/2000 ms, FA = 70^o^, FOV = 200 mm) were obtained using echo planar acquisition. Image volumes consisted of 28 5-mm thick axial slices with 3.125 × 3.125 mm^2^ pixels. T1-weighted anatomical volumes were obtained using SPGR (TE/TR = 2.6/2000 ms, FOV = 256 mm, slice thickness = 1 mm). These data were originally presented in (Grady *et al.*^25^).

Subject brain volumes were nonlinearly co-registered to a common anatomical template, created from a group-average anatomical reference; this was obtained from an iterative optimization procedure that is robust to inter-subject anatomical variability^48^. Functional data were corrected for slice-timing offsets using AFNI (afni.nimh.nih.gov/afni) and motion corrected using AIR (bishopw.loni.ucla.edu/AIR5). Additional preprocessing consisted of spatial smoothing with a FWHM = 7 mm Gaussian kernel and regressing out white matter; linear detrending was also performed to control for residual motion and scanner drift effects.

Subjects performed 4 task runs (total duration of 300TR = 600 s, for each run), in which blocks of 4 different stimuli were presented twice in each run, interleaved with fixation blocks; for full details, see^25^. The visual stimuli consisted of band-pass filtered white noise patches with different center frequencies; for all tasks, when subjects detected a “target” they pressed 1 of 3 buttons to indicate where the stimulus appeared. During experimental planning, all tasks were designed to have an average accuracy level of approximately 80% across subjects. The stimulus types include:

**Reaction Time (RT)**: When the participant detected a target image on a screen, they pressed the corresponding button as quickly as possible.

**Delayed Match (DMS)**: Test of working memory. A target stimulus was presented and then removed from the screen, followed by a 2.5 seconds blank-screen delay. Three stimuli were presented and the participant pressed the button corresponding to the target.

**Attentional Cueing (ATT)**: The participant pressed the button corresponding to the target image, following a brief directional cue.

**Perceptual Matching (PMT)**: The participant was presented a target in the upper portion of the screen, and three stimuli on the lower portion; they pressed the button corresponding to the one of three stimuli on the lower portion that matched the target.

During each of the 4 experimental runs, tasks were presented in 2 × (~20 TR) blocks per run per condition (8 blocks total), where the order of task blocks was randomly permuted for each subject and run.

#### SART and TMT Dataset

This dataset included 27 young, healthy adults (median age 24, range 20 to 33; 16 female), and 22 older, healthy adults (median age 68, range 61 to 82; 12 female). Subjects were confirmed right-handed using the Edinburgh Handedness Inventory^49^, and screened for cognitive and neurological deficits, by self-report and using the Mini-Mental Status Examination^50^. Images were acquired on a 3T MR scanner (MAGNETOM Tim Trio, VB15A software; Siemens AG, Erlangen, Germany), with a 12-channel head coil. A T1-contrast anatomical scan was obtained (oblique-axial 3D MPRAGE, 2.63/2000/1100 ms TE/TR/TI, 9° FA, 256 × 192 matrix, 160 slices per volume, voxel dimensions 1 × 1 × 1 mm3), followed by BOLD fMRI (2D GE-EPI, 30/2000 ms TE/TR, 70^o^ FA, 64 × 64 matrix, 30 slices per volume, voxel dimensions 3.125 × 3.125 × 5 mm^3^). These data were previously presented in (Churchill *et al.* 2012, 2013).

Brain masks were obtained for EPI and T1 data, using FSL’s Brain Extraction Tool (ver. 5.5; www.fmrib.ox.ac.uk/fsl/), and warped subject EPI data into MNI template space using FSL’s *flirt* package, with the “avg152T1_brain.nii” as our reference volume. We performed rigid-body motion correction using AFNI *3dvolreg*, and registered to the within-run volume of minimum displacement. We identified high-motion outlier spikes using a statistically-driven procedure, and replaced outliers with interpolated values from neighbouring volumes^51^; a median of 2 timepoints replaced per run (range 0 to 5). We then performed slice-timing correction with AFNI *3dTshift*, and spatial smoothing with an isotropic 6 mm Gaussian kernel with AFNI *3dmerge*. We also performed linear detrending and corrected for residual motion by regressing out the first 2 PCs of the rigid-body motion parameter estimates (accounting for > 85% of head motion variance), to provide strong control against head motion confounds.

Both tasks were acquired as part of a brief fMRI battery, designed for assessment of stroke patients. The tasks included a block-design fMRI adaptation of the Trail-Making Test (TMT) and a rapid event-related Sustained Attention to Response Task (SART):

**Trail-Making Test (TMT)**: The task consisted of two task conditions: TaskA, in which numbers 1–14 are pseudo-randomly displayed on a viewing screen, TaskB, in which numbers 1–7 and letters A-G are displayed. Subjects used an MRI-compatible writing tablet and stylus to draw a line connecting items in sequence (1-2-3-4-…) or (1-A-2-B-…), connecting as many as possible for a 10TR (20 sec.) block while maintaining accuracy. A Control stimulus was presented after each task block, in which subjects trace a line from the center of the screen to a randomly placed dot and back over 2 s, repeated 10 times. Each task run was 80 scans (160 sec.) in length, consisting of two 4-block epochs of TaskA–Control-TaskB-Control conditions.

**Sustained Attention to Response Task (SART)**: This task was a fast event-related GO-NOGO design. The set of integers 1-9 were presented in random order on the screen, followed by a masking image. Stimuli were presented for 250 ms, while the mask was shown for a randomized interstimulus interval, of mean 1250 ± 210 ms. Subjects were asked to respond to all integers except ‘3’ (the NOGO stimulus) using the MRI-compatible writing tablet, by touching the stylus to the tablet surface. A single run consisted of 100 presented digits, with 75 GO stimuli and 25 NOGO stimuli, in randomized order, with 76 scans per run (152 sec.).

We acquired two runs of each task during a single test session, separated by approximately 12 minutes, during which they performed other tasks.

#### VWMT Dataset

for this task, we used healthy control data from a study of the effects of breast cancer on working-memory performance^39^. This included 30 healthy adult women (median age 52, range 36 to 71), who were pre-screened for intact cognitive function using the Mini Mental Status Examination^50^ and absence of clinical depression using the Patient Health Questionnaire^52^ based on DSM-IV-TR criteria. All participants were right-handed and met the MRI screening criteria. Images were acquired on a GE Signa 3 Tesla scanner equipped with a standard quadrature head coil. A 124-slice high-resolution T1-weighted was collected (SPGR. 1.8/9 ms TE/TR, 15^o^ FA, FOV = 25–26 cm, slice thickness = 1.2 mm) and BOLD T2* weighted images were acquired (spiral sequence, 30/1500 ms TE/TR, 70^o^ FA, 24 cm FOV, 25 slices per volume, voxel dimensions 3.75 × 3.75 × 5 mm).

Each SPGR was corrected for signal in-homogeneity and skull-stripped using FSL’s Brain Extraction Tool. These images were then normalized with SPM5 (Wellcome Department of Cognitive Neurology, London); the normalization parameters for warping to the standard MNI template were recorded and applied to the functional images. Functional images were corrected for differences in slice timing using 4-point sinc-interpolation, corrected for head movement using FSL’s MCFLIRT. Outliers were removed and interpolated from neighbouring volumes using cubic splines to avoid discontinuities in BOLD signal; a median of 2 timepoints replaced per run (range 0 to 5). We then performed slice-timing correction with AFNI *3dTshift*, and spatial smoothing with an isotropic 6 mm Gaussian kernel with AFNI *3dmerge*. We also performed linear detrending and corrected for residual motion by regressing out the first 2 PCs of the rigid-body motion parameter estimates (which account for > 85% of head motion variance), to provide strong control against head motion confounds.

**Verbal Working Memory Task (VWMT)**: Participants performed a verbal working memory task (VWMT) during fMRI scanning. For each trial in the VWMT, participants were presented with a set of 4 letters for 1500 ms. Following a 3000 ms delay interval, they were presented with a “probe” letter for 1500 and asked whether it was a member of the current memory set. Each subject was presented with 192 trials, acquired over 4 scanning runs of 285 TR (427.5 s); inter-trial Intervals were jittered, ranging between 1500 ms and 9000 ms.

We acquired four task runs during a single test session, for which we analyzed the first 2 runs.

### Power Spectral Density

For scale-free processes, the power spectral density (PSD) exhibits a power-law relationship with frequency (*f* ), given by the equation: *P*(*f* ) ∝ *f*^*−β*^. This scaling exponent *β* is related to the Hurst exponent by *β* = 2H−1. This approach was compared to Detrended Fluctuations Analysis (DFA) in Fig. 2 and in supplementary Figures S1–S4. To estimate *H* at each voxel, we computed the PSD via Hanning-windowed Welch estimator, analyzed in time windows of the same length as those used in DFA (see *Selection of Scaling Ranges for fMRI Data* below). For SART, TMT and VWMT, this was done on 50-TR windows, whereas the briefer MTAS task blocks were analyzed in 20-TR windows. For all datasets, we estimated *β* based on a linear fit of the plot of log(*f* ) vs. log(P(*f* )) for the frequency range between 0.02 and 0.20 Hz, in order to reduce the impact of low-frequency signal confounds.

### Detrended Fluctuations Analysis

For our main analyses and results, we measured scale-free signal in fMRI using DFA^53^. This is an efficient estimator of the Hurst exponent that, unlike power spectral density (PSD) approach, is intrinsically robust to signal non-stationarity and low-frequency confounds. For a voxel time-course ***x***(*t*) (1 ≤ *t* ≤ *T*), we first transform it into an unbounded random walk, by computing the integrated time-series 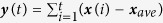. We then subdivide ***y***(*t*) into a series of time-windows of equal length *n*, and estimate a least-squares linear regression fit per window; this provides a fitted linear estimate at every point in the timeseries, denoted 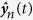, for a window size *n*. Next, we compute the root-mean-square (RMS) magnitude of fluctuations on the detrended data:


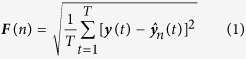


The RMS fluctuation ***F***(*n*) is computed over a range of window sizes *n*, and we plot the linear fit of log(n) vs. log(***F***(*n*)). The slope of this fitted line, which corresponds to the Hurst exponent *H*, measures the degree of fractalness for voxel time-series ***x***(*t*). A slope of *H* = 0.5 indicates a random walk with no long-range correlations; and increasing slope denotes greater power-law scaling, up to *H* = 1, which corresponds to long-range, fractal scaling.

The DFA approach can be considered a modification of standard PSD techniques, which seek to find the scaling exponent *β* based on the relation *P*(*f* ) ∝ *f*^*−β*^, by finding the slope of linear fit for the log-scale plot of *P*(*f* ) as a function of *f*. As established by Heneghan & Darby^54^, for a sampling interval of *δ* (sec), the mean squared fluctuations at window size *n* is equal to:


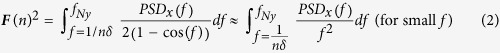


Where *PSD*_*x*_(*f *) is the spectral power of the raw time-series ***x*** at frequency *f*, and the Nyquist frequency *f*_*Ny*_ = 1/2*δT*. Thus, RMS fluctuations are a function of total spectral power for *f* > 1/*nT*, weighted by a factor 1/*f*^ 2^, so that lower frequencies near 1/*nT* contribute most to RMS variability at time-scale *n*.

For a “mono-fractal” scale-free process, scaling phenomena are fully described by a single exponent *H*, and asymptotically invariant for the range of window sizes *n* that are analyzed by DFA. For such processes, signal autocorrelations persist across all measured time-scales. However, in real data, “cross-overs” may be observed, i.e., transition points where *H* changes as a function of *n*. This may be due to the presence of different scaling regimes, or trends in data that are not sufficiently controlled by the linear detrending step. Serial autocorrelation in fMRI data, such as AR(1) processes, have a characteristically short time-scale dependency. They will manifest as a DFA plot with a high scaling exponent for short time windows within one order of magnitude, and a transition beyond this point to a lower scaling exponent where the AR(1) influence rapidly decays^55^. To determine whether such confounds are present, it is critical to examine the goodness of fit for the DFA regression line, over all examined time-scales. This was examined using the coefficient of determination (*R*^2^), summarized in-text at the beginning of the Results section and displayed in supplementary Figure S1.

### Selection of Scaling Ranges for fMRI Data

For all datasets except MTAS, we performed DFA for *n* ranging from 50 TRs (75 sec for VWMT, 100 sec for SART and TMT), which excludes possible low-frequency confounds below 0.01 Hz, to 3 TRs (4.5 sec for VWMT, 6 sec for SART and TMT), which is the shortest interval where linear regression gives a non-zero residual. For the MTAS dataset, DFA was performed on *n* ranging from 20 TRs (40 sec), which is the maximum block size, to 3 TRs (6 sec). For all analyses, we sampled window sizes uniformly with a log-scale across this range, to ensure that no specific time-scale dominated the linear curve fitting. The finest possible sampling of discrete window sizes without duplication was 13 values of *n* across this interval for VWMT, SART and TMT, and 11 values of *n* for MTAS.

For the jittered event-related SART and VWMT tasks, DFA was performed directly on each voxel time-series, for each subject and task run. For TMT, the presence of a periodic block paradigm may lead to spurious “cross-over” effects in the DFA scaling curve at the central frequency of the design. We therefore examined the DFA regression fit (supplemental Fig. S1); based on high degree of fit, which was comparable to the aperiodic SART task of similar length, we concluded that it was appropriate to directly apply DFA to this dataset. For all MTAS tasks, we performed DFA separately for each 20-TR task block and then averaged across the 8 blocks from 4 runs per task. The DFA analyses produced a single brain map of *H* values, for each subject and task condition. In the section *Impact of Scaling Window Size* below, we examined the stability of the shorter time-window approach used for MTAS data.

### Effects of Task Complexity

The MTAS dataset was used to evaluate the effects of task complexity on fractal scaling, as this battery consisted of a set of tasks with similar goals and visual stimuli, but varying levels of task difficulty. For each subject and task, we computed a mean *H* map across all blocks and runs. We then performed a Partial Least Squares (PLS) analysis on the *H* maps of the 4 tasks [see^56^ *et al.*, 2011 for an overview of PLS]. This was done by computing the average *H* maps for the 4 different tasks ***H***_PMT_, ***H***_DMS_, ***H***_ATT_ and ***H***_RT_. We concatenated these vectors into a single matrix **H**_cat_ = [***H***_PMT_, ***H***_DMS_, ***H***_ATT_, ***H***_RT_], normalized by z-scoring across rows, and computed the singular value decomposition ***UΛV***^T^ = **H**_cat_. This produces a ***U*** matrix containing spatial brain patterns (brain saliences), whose modulation across tasks is given by vectors in ***V*** (task saliences). We computed confidence bounds on values in ***U*** and ***V*** by Bootstrap resampling on subjects (1000 iterations). These were used to generate Bootstrap ratios on brain voxels in ***u***_i_, reflecting significant brain regions; maps were thresholded at False-Discovery Rate (FDR) of 0.05 to correct for multiple comparisons. We also generated 95% confidence intervals (CIs) on the task saliences ***v***_i_. For these analyses, we only retained the one latent variable set (***u***_i_ ***v***_i_) that had significant brain and task saliences after FDR = 0.05 correction.

### Effects of Task Novelty

We used data from SART and TMT (young subjects), and VWMT (all subjects) to evaluate the effects of task novelty (i.e. task exposure) on fractal scaling. For SART and TMT, we computed an *H* map for each subject, for run-1 and run-2. We then measured the mean change Δ*H* between task runs, averaged across subjects, for each brain voxel. We measure the reliability of between-run Δ*H* by Bootstrap resampling on subjects (i.e. resampling with replacement), and computing the mean Δ*H* for the Bootstrap sample, repeated for 1000 resampling iterations. We obtain empirical *p*-values by computing the fraction of resamples with mean Δ*H* > 0, thresholded at FDR = 0.05; the Bootstrap ratios for significant brain regions were displayed. For VWMT, we analyzed the 4 different task runs by computing the average *H* maps for the 4 different task runs ***H***_1_, ***H***_2_, ***H***_3_ and ***H***_4_, and the performing the Bootstrapped PLS procedure outlined in the previous section, to obtain spatial saliences ***u***_i_ and run saliences ***v***_i_. For these analyses, we only retained the one latent variable set (***u***_i_ ***v***_i_) that had significant brain and task saliences after FDR = 0.05 correction.

### Effects of Aging

We used data from SART, TMT and VWMT to evaluate the effects of aging on fractal scaling. For each subject, we computed a mean *H* map across runs. For SART and TMT, we plotted maps of the mean Δ*H* between older and younger groups for each task. We estimate regions of significant change by Bootstrap resampling on subjects within the young and old groups and computing the mean Δ*H* between older and younger Bootstrap samples, repeated for 1000 resampling iterations. This is used to compute empirical *p*-values and threshold at FDR = 0.05, in the same manner as described in the previous section. For VWMT, we examined a more continuous change, as subject ages ranged between 36 and 71 years. To test for a continuous change in *H* with age, we performed an additional PLS analysis. In this case, we performed a simple correlation PLS, as there is only one non-fMRI variable (i.e. age). We therefore concatenated all of the individual subject *H* maps into matrix **H**_cat_, normalized by z-scoring across rows, and concatenated all subject ages into the vector ***y***_cat_. We then computed the map of changes in *H* related to age ΔH = **H**_cat_***y***_cat_. Note that this is equivalent to ridge regression of ***y*** onto ***H*** with an infinitely large ridge penalty. We obtained bootstrap ratios on ΔH, by Bootstrap resampling on subjects with an FDR = .05 correction for multiple comparisons. We also plotted subject scores **H**_cat_^T^ΔH, showing how expression of ΔH is related to age.

### Impact of Scaling Window Size

For all analyses except task complexity, BOLD scaling phenomena were examined across a fixed range of 3 to 50 TRs; due to the shorter block lengths of the MTAS data, *H* was computed for task complexity analyses from 3 to 20 TRs (40 sec). In order to validate this approach, and determine the robustness of DFA to shorter time-windows, we examined the consistency of findings as a function of maximum window size *n*_max_ for the representative SART dataset. We recalculated the *H* maps for *n*_max_ ranging from 50 TRs to 20TRs (100 to 40 sec) in steps of 5, and averaged *H* maps in the same manner as for with the MTAS data. We examined the overall stability of the *H* maps as a function of *n*_max_, by plotting the correlations between subject *H*-maps obtained using *n*_max_ = 50 TR and *H*-maps obtained from shorter *n*_max_ values. In addition, we re-analyzed task novelty effects (run-1 vs. run-2) and aging (old vs. young) as a function of *n*_max_ and plotted maps of significant brain regions, to determine if there was a consistent decrease in sensitivity to these sources of cognitive effort for shorter *n*_max_ values.

### Relation to BOLD Variability and Functional Connectivity

In this section, fractal scaling was compared against widely-used alternative metrics of BOLD dynamics, for the representative SART dataset. The standard deviation of BOLD signal (SD_BOLD_) was computed as a measure of “total variability”, which is thought to reflect the dynamic range of neurovascular function at each voxel, and has previously shown utility in studies of aging and cognitive functioning^34^. Because variance is potentially highly sensitive to motion and physiology noise, it was computed on data in the frequency range of 0.01 to 0.08 Hz by (a) detrending the data, and (b) applying a low-pass Butterworth filter with pass-band (>50% attenuation) at 0.08 Hz and stop-band (<1% attenuation) at 0.10 Hz. This filtered signal was then computed as the % variability relative to the voxelwise mean.

Global functional connectivity (*Gconn*) was also evaluated, as a measure of total integrative brain function, which has been well-studied^57^,^58^,^59^. Brain maps were obtained by computing, for each voxel, the average correlation of the BOLD timeseries with all other brain voxels that have positive correlations. This was used as a way to measure the overall connection strength with other voxels, without having ambiguities due to negative and positive correlations cancelling out. We computed *Gconn* on data without low-pass filtering in order to ensure the same information content was used to derive both this measure and estimation of *H*.

We plotted the average *H*, SD_BOLD_ and *GConn* maps, along with pairwise scatterplots of median voxel values computed across subjects, displayed separately for run-1 and run-2 (young subjects only), and for young and old subjects (averaging across runs). To evaluate the consistency of brain maps provided by these BOLD metrics, we also reported the mean non-parametric Spearman correlation between each pair of measures, along with the bootstrapped 95% confidence intervals (95%CIs). In addition, we evaluated the relative change associated with effort for each BOLD metric, using the non-parametric Kolmogorov-Smirnov (K-S) test to determine if there was a significant change in the distribution of median voxel values for run-1 vs. run-2 (task novelty) and old vs. young groups (aging). We compared the relative size of these effects for *H*, SD_BOLD_ and *Gconn*, by bootstrap resampling on subjects (1000 iterations) and obtaining empirical p-values based on the fraction of resamples showing a consistent different in K-S test statistic, with the 95%CIs also reported.

## Additional Information

**How to cite this article**: Churchill, N. W. *et al.* The suppression scale-free fMRI brain dynamics across three different sources of effort: aging, task novelty and task difficulty. *Sci. Rep.* **6**, 30895; doi: 10.1038/srep30895 (2016).

## Supplementary Material

Supplementary Information

## Figures and Tables

**Figure 1:**
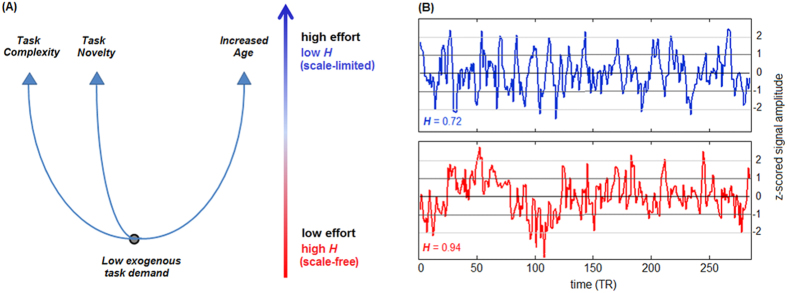
Plots depicting (**A**) the central hypothesis of the paper, and (**B**) fractal scaling examples in fMRI. (**A**) schematic illustrating the key hypothesis of this paper: *effort is inversely related to fractal scaling*. For low task effort, the brain is in a highly scale-free “ground” state (depicted by the black disk). Numerous factors increase cognitive effort (and decrease *H*), including task complexity, task novelty, and aging. This is analogous to the “potential energy wells” that are often employed in physics diagrams. (**B**) BOLD timecourse for two subjects from the Verbal Working Memory Task (VWMT), based on an 8-voxel seed in the posterior cingulate, with Hurst exponents of *H* = 0.72 (moderately fractal) and *H* = 0.94 (highly fractal). We observe smooth, highly autocorrelated time-series for the more fractal red curve.

**Table 1:** Performance measures for different task conditions of the Multi-Task Assessment battery (MTAS).

Task Condition	Response Time (ms)	Percent Correct
Perceptual Matching (**PMT**)	1538 (±386)	83.5 (±3.7)
Delayed Match (**DMS**)	1150 (±229)	88.6 (±6.1)
Attentional Cueing (**ATT**)	1191 (±262)	84.5 (±5.7)
Reaction Time (**RT**)	506 (±85)	97.9 (±3.0)

Mean performance measures are reported across subjects, with ± standard deviation.

**Table 2:** Performance measures for different experimental tasks, comparing run-1 (more novel) vs. run-2 (more familiar).

Task Condition	Response Time (ms) Run-1	Response Time (ms) Run-2	Percent Correct Run-1	Percent Correct Run-2
Trail-Making Test (**TMT**)	1620 (±345)*	1310 (±318)*	—	—
Sustained Attention to Response Task (**SART**)	407 (±85)	402 (±96)	92.8 (±5.8)*	94.5 (±5.5)*
Verbal Working Memory Task (**VWMT**)	1188 (±193)*	1142 (±207)*	94.2 (±5.0)	94.9 (±5.8)

We report mean performance measures across subjects, ± standard deviation. A “*” indicates a significant difference between runs, at p < 0.01, paired Wilcoxon test. For VWMT, we chose runs 1 and 2, out of the 4 available runs.

**Table 3:** Performance measures for different experimental tasks, comparing young vs. old subjects.

Task Condition	Response Time (ms) Young	Response Time (ms) Old	Percent Correct Young	Percent Correct Old
Trail-Making Test (**TMT**)	1465 (±257)*	1938 (±444)*	—	—
Sustained Attention to Response Task (**SART**)	405 (±83)*	423 (±83)*	93.7 (±5.4)*	90.9 (±14.2)*
Verbal Working Memory Task (**VWMT**)	1074 (±137)*	1190 (±151)*	97.4 (±3.1)*	93.3 (±5.5)*

We report mean performance measures across subjects, ± standard deviation. A “*” indicates a significant difference between age groups, at p < 0.01, Wilcoxon rank-sum test. For VWMT, data were split into the 10 youngest and 10 oldest subjects; “Young” has a median age 41 (range 36 to 46) and “Old” has a median age 60 (range 55 to 71), a much smaller age difference. We performed significance testing on the difference between these two groups.

**Table 4:** Correlations between Hurst exponent (H), standard deviation of BOLD signal (SD_BOLD_) and global connectivity (Gconn) brain maps.

	corr(H, SD_BOLD_)	corr(H, Gconn)	corr(SD_BOLD_, Gconn)
**A**.			
Run-1 (young)	0.60 [0.54, 0.66]	0.54 [0.46, 0.63]	0.34 [0.27, 0.40]
Run-2 (young)	0.67 [0.62, 0.71]	0.62 [0.54, 0.69]	0.38 [0.29, 0.46]
**B**.			
Young (run1 and 2)	0.66 [0.62, 0.71]	0.63 [0.56, 0.70]	0.39 [0.31, 0.45]
Old (run1 and 2)	0.42 [0.30, 0.51]	0.47 [0.34, 0.56]	0.19 [0.11, 0.28]

Results are shown for representative SART dataset, for brain maps averaged across subjects for (A) young adults, comparing Run-1 vs. Run-2, and (B) for both runs, comparing Young vs. Old cohorts. The mean bootstrapped correlation is reported for each, along with 95% confidence intervals (95%CIs).
