# Supplementary material for: The suppression of scale-free fMRI brain dynamics across three different sources of effort: aging, task novelty and task difficulty
Source: Sci Rep. 2016 Aug 8;6:30895. doi: 10.1038/srep30895 (PMC4976369; doi:10.1038/srep30895)
Supplement: Supplementary Information [file srep30895-s1.pdf]

Supplementary Information for:

The suppression of scale-free fMRI brain dynamics across three different sources of effort: aging, task novelty and task difficulty

Nathan W. Churchill[1,2], Robyn Spring[1], Cheryl Grady[1], Bernadine Cimprich[3], Mary K. Askren[4,5], Patricia A. Reuter-Lorenz[4], Mi Sook Jung[6], Scott Peltier[7], Stephen C. Strother[1,2,8], Marc G. Berman[9]

Affiliations:

[1] Rotman Research Institute, Baycrest Hospital.
[2] Department of Medical Biophysics, University of Toronto.
[3] School of Nursing, University of Michigan.
[4] Department of Psychology, University of Michigan.
[5] Department of Psychology, University of Washington.
[6] College of Nursing, Chungnam National University.
[7] Biomedical Engineering, University of Michigan.
[8] Institute of Medical Science, University of Toronto.
[9] Department of Psychology, University of Chicago

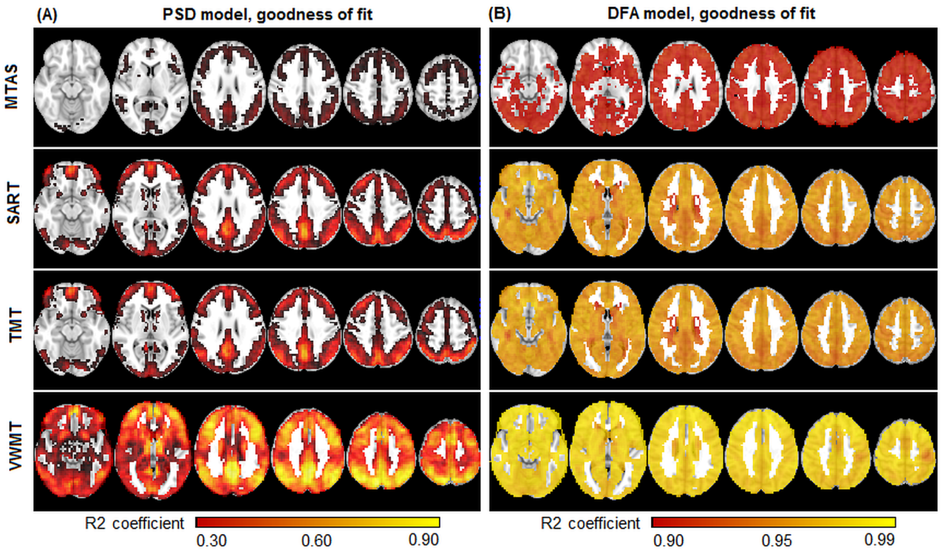

Figure S1: goodness of fit for power spectral density (PSD) and detrended fluctuations analysis (DFA) models, for each task. Maps depict the coefficient of determination (R^2), quantifying goodness of fit for the PSD and DFA models, used to estimate the Hurst exponent *H*. The R^2 values are averaged across subjects for each task. Results for MTAS data are also averaged across all conditions.

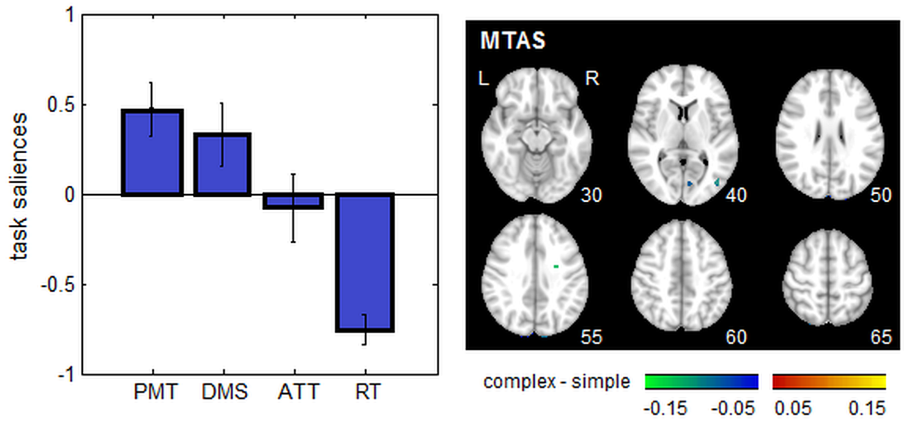

Figure S2: brain regions showing decreased Hurst exponent (*H*) going from simple to complex tasks, for power spectral density (PSD) estimator. Results of a Partial Least Squares analysis are shown for four different tasks obtained from a Multi-Task Assessment battery, reaction time (RT), attentional cueing (ATT), delayed match to sample (DMS) and perceptual matching (PMT). We display results of the first component, which accounts for 44.3% total covariance (significant at $p<0.001$, permutation testing). (left) plots task saliences with Bootstrapped 95% CI errorbars, and (right) plots associated significant bootstrap ratio values in the brain at a False Discovery Rate threshold of 0.05, indicating widespread negative change in Hurst exponent, associated with the more complex DMS and PMT conditions. A liberal cluster-size threshold of >3 contiguous voxels was also applied to improve image interpretability.

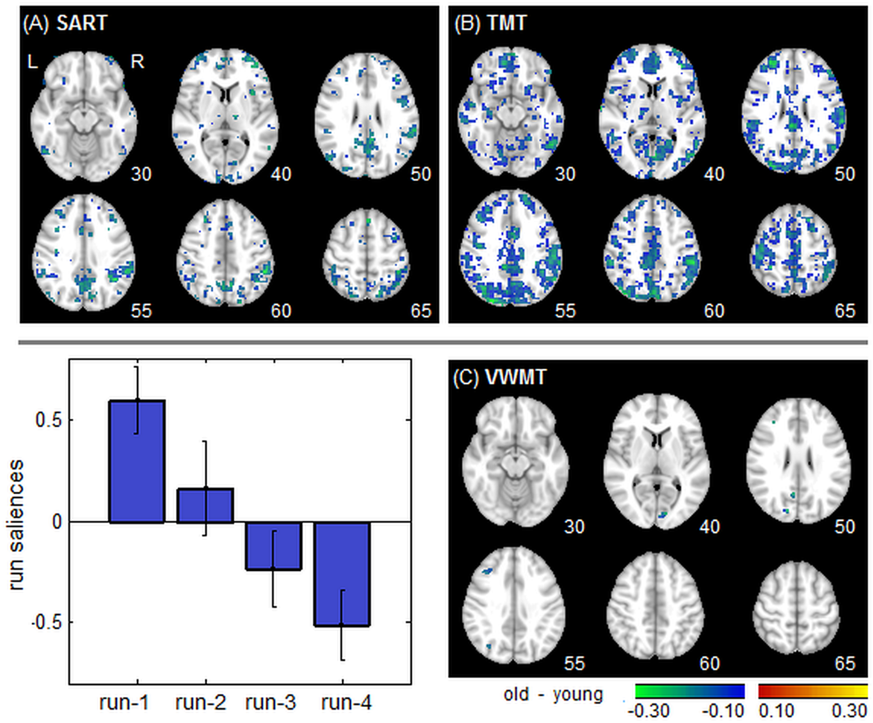

Figure S3: brain regions showing decreased Hurst exponent (*H*) going from run-2 to run-1 of a task, for power spectral density (PSD) estimator. Panels (A,B) show results of pairwise testing of Hurst exponent in run-1 vs. run-2, for TMT = Trail-Making Test (block design) and SART = Sustained Attention to Response Task (fast event-related). (C) Results of a Partial Least Squares analysis of 4 runs of a VWMT = Verbal Working Memory Task (slow event-related). We display results of the first component, which accounts for 54.0% total covariance (significant at $p<0.001$, permutation testing), showing decreased Hurst exponent (negative Bootstrap ratios) is associated with later task runs. All Bootstrap ratio maps are corrected for multiple comparisons at FDR=.05 threshold; run saliences have 95% CI errorbars. A liberal cluster-size threshold of >3 contiguous voxels was also applied to improve image interpretability.

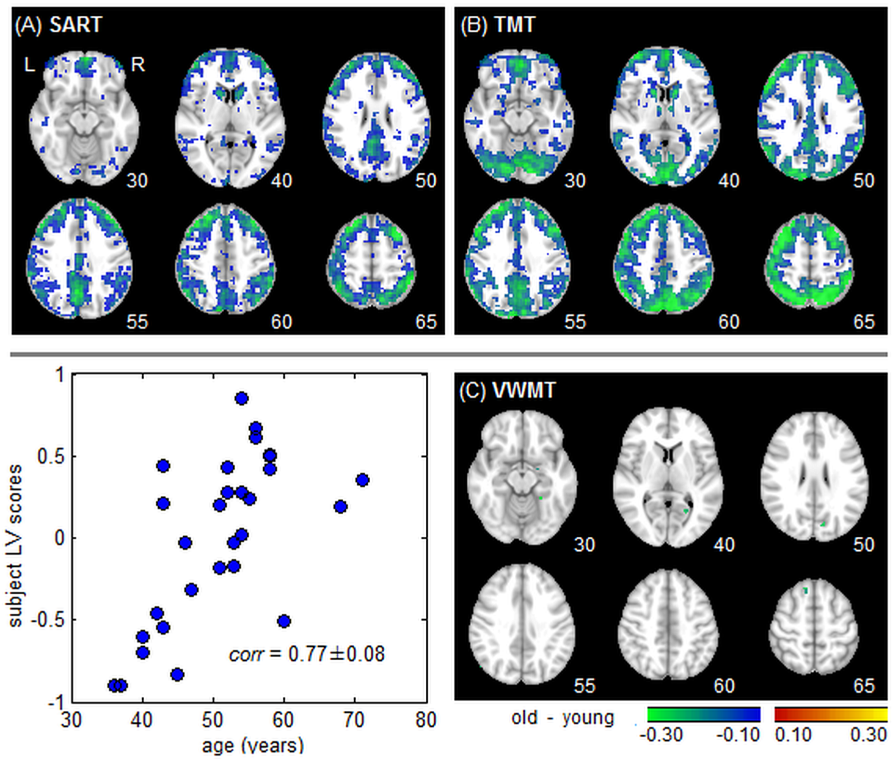

Figure S4: brain regions showing decreased Hurst exponent (*H*) going from older to younger subjects, for power spectral density (PSD) estimator. Panels (A,B) show results of pairwise testing of Hurst exponent in young vs. old, for TMT = Trail-Making Test (block design) and SART = Sustained Attention to Response Task (fast event-related). (C) Results of a Partial Least Squares analysis of Hurst exponent against age for VWMT = Verbal Working Memory Task (slow event-related), showing decreased Hurst exponent (positive Bootstrap ratios) is associated with age. All Bootstrap ratio maps are corrected for multiple comparisons at FDR=.05 threshold. A liberal cluster-size threshold of >3 contiguous voxels was also applied to improve image interpretability.
